# Improving early liver metastasis detection in colorectal cancer using a weighted ensemble of ResNet50 and swin transformer: a KHCC study

**DOI:** 10.3389/fdata.2025.1700292

**Published:** 2026-01-12

**Authors:** Ahmad Nasayreh, Hasan Gharaibeh, Rula Al-Qawabah, Azza Gharaibeh, Bayan Altalla, Iyad Sultan

**Affiliations:** 1 Artificial Intelligence Office, King Hussein Cancer Center, Amman, Jordan; 2 Department of Radiology, King Hussein Cancer Center, Amman, Jordan; 3 Office of Scientific Affairs and Research, King Hussein Cancer Center, Amman, Jordan

**Keywords:** colorectal cancer, deep learning, ensemble learning, liver metastasis, transformer

## Abstract

Colorectal cancer represents the third most diagnosed malignancy globally, with liver metastasis occurring in approximately 50–60% of patients following initial treatment. Current surveillance strategies utilizing carcinoembryonic antigen monitoring and interval cross-sectional imaging demonstrate significant limitations in early hepatic recurrence detection, often identifying disease at advanced, unresectable stages. This study addresses the critical research gap in AI-driven surveillance frameworks by developing a novel ensemble deep learning model for early liver metastasis prediction in colorectal cancer patients. The methodology employed six state-of-the-art architectures including ResNet50, MobileNetV2, DenseNet121, CNN-LSTM, and Swin Transformer as feature extractors through transfer learning, followed by weighted soft voting ensemble learning combining the top-performing models. The framework was evaluated on a comprehensive dataset of 1,628 medical images from colorectal cancer patients, with rigorous statistical validation using Friedman and Wilcoxon signed-rank tests. Results demonstrated that the ensemble model combining ResNet50 and Swin Transformer achieved superior performance with 75.48% accuracy, 79.0% sensitivity, 73.6% specificity, and 0.8115 AUC, representing statistically significant improvements over all individual architectures. The ensemble approach successfully addressed the challenging nature of the dataset where multiple state-of-the-art models achieved near-random performance, demonstrating the effectiveness of architectural diversity in medical image analysis. The clinical impact of this work extends to enhancing early detection capabilities that could increase patient eligibility for curative interventions, with balanced diagnostic performance suitable for surveillance applications. The computationally efficient framework requires only 0.39 s per image inference time, making it feasible for integration into existing clinical workflows and potentially improving outcomes for colorectal cancer patients through earlier identification of hepatic recurrence.

## 1 Introduction

Colorectal cancer (CRC) remains a leading cause of global cancer morbidity and mortality, accounting for nearly one in ten cancers and close to one in ten cancer deaths worldwide according to the most recent GLOBOCAN 2022 update (Bray et al., 2024). In 2022, approximately 1.93 million new CRC cases and 904,000 CRC deaths were estimated, underscoring a substantial and growing burden that contributes significantly to worldwide cancer incidence and mortality. Contemporary summaries from international agencies similarly emphasize CRC as the third most diagnosed malignancy and the second leading cause of cancer death globally, reflecting persistent gaps in early detection and surveillance (Siegel et al., 2024). Broader analyses of gastrointestinal cancers indicate that GI malignancies, including CRC, account for roughly one-third of global cancer deaths, further highlighting CRC's central role in worldwide cancer outcomes and the need for improved strategies across the care continuum (Tian et al., 2024). Hepatic recurrence represents a major clinical challenge among CRC survivors, as the liver is the most common site of metastasis and relapses following initial treatment, including resection of Colorectal Liver Metastases (CRLM) (Patel et al., 2023). Despite advances in systemic therapy and hepatic surgery, overall recurrence after initial liver resection occurs in approximately 50%−60% of patients, with intrahepatic recurrence reported in 20%−47%, patterns consistent with occult micro metastatic disease not visible on baseline imaging. Early recurrence often within 6 months of hepatectomy occurs in a considerable fraction of patients and is associated with adverse tumor and treatment factors such as poor differentiation, billboard involvement, margin positivity, major hepatectomy, and postoperative complications, all of which portend inferior long-term survival (Guo et al., 2024). Although repeat liver resection can improve survival in selected patients with isolated intrahepatic relapses, the therapeutic window narrows quickly when detection is delayed, making earlier identification of hepatic recurrence pivotal for enabling potentially curative interventions.

Current post-treatment surveillance strategies for CRC anchored in serial Carcinoembryonic Antigen (CEA) monitoring and interval cross-sectional imaging with Computed Tomography (CT) and Magnetic Resonance Imaging (MRI) are codified across major society guidelines but show important limitations for early hepatic recurrence detection. CEA exhibits variable performance, with sensitivity and specificity influenced by threshold selection, and a considerable proportion of CEA-detected relapses are unresectable at the time of identification, implying delayed capture of actionable disease. CT surveillance, commonly recommended at semi-annual to annual intervals for up to 5 years after curative treatment, improves detection when combined with CEA but remains constrained by interval scheduling, radiation exposure, small lesion conspicuity, and inter-reader variability, all of which limit sensitivity for very early hepatic relapse. Liver MRI offers superior soft-tissue contrast and lesion characterization compared with CT, yet resource demands, variability in access, and inconsistent adherence to intensive schedules reduce its utility for widespread early detection in routine practice (Lauretta et al., 2023). Parallel advances in deep learning and artificial intelligence (AI) have transformed medical image analysis, with accumulating evidence that AI can match or exceed expert performance by leveraging high-dimensional features beyond human perception. In CRC imaging specifically, recent systematic analyses of AI models trained on radiologic data report high diagnostic accuracy for predicting distant metastasis, with pooled estimates indicating strong sensitivity and specificity that could translate into earlier detection relative to conventional reads. The capability of deep neural networks to detect subtle textural and morphological signatures that preceded overt radiologic progression suggests an opportunity to identify hepatic recurrence at smaller lesion sizes, potentially increasing eligibility for curative resection or ablation Nonetheless, translation to routine surveillance has been uneven due to study heterogeneity, non-standardized imaging protocols, and a paucity of prospective, real-world validation embedded within longitudinal follow-up pathways (Zhang et al., 2022). A critical research gap persists there is not widely adopted, validated, AI-driven surveillance framework specifically tailored to early hepatic recurrence detection in CRC that integrates with guideline-based follow-up and demonstrates improvements in time-to-detection, respectability, and survival. Existing guidelines largely specify the cadence and modalities of surveillance but do not incorporate AI-enhanced image analysis or standardized multimodal fusion of biomarkers and imaging features into routine workflows, and most AI studies have not been designed to target early hepatic recurrence as a primary, clinically actionable endpoint in prospective surveillance settings. In view of the global burden and the prognostic importance of timely detection, a rigorously validated AI framework focused on early hepatic relapses would address a consequential unmet need in oncologic surveillance (Sung et al., 2021).

Accordingly, this study proposes an advanced deep learning model for early hepatic recurrence detection in patients with CRC, integrating multiphasic imaging with clinically available biomarkers to enhance sensitivity while maintaining acceptable specificity within standard surveillance intervals. By targeting sub-radiologic or subtly radiologic hepatic changes predictive of imminent recurrence, the model aims to shift detection earlier in the disease trajectory, thereby increasing the proportion of patients eligible for potentially curative local therapies and improving downstream outcomes. Framed against the rising global burden of CRC and the limitations of existing surveillance approaches, this AI-enabled framework seeks to deliver an innovative and impactful contribution to oncology and precision medicine through smarter detection strategies and optimized care pathways.

The main contribution in this study is the following:

1. First validated ensemble framework introduces a statistically validated ensemble methodology for liver metastasis detection, addressing fundamental gaps in current AI-driven diagnostic systems beyond single-model approaches.
2. Novel King Hussain Cancer Center (KHCC) dataset presents a comprehensive real-world cancer imaging dataset from KHCC, providing standardized benchmark data for hepatic metastasis detection research.
3. Multi-architecture integration develops a hybrid approach combining CNN feature extraction (ResNet50) with transformer-based global modeling (Swin Transformer) to optimize sensitivity and specificity metrics simultaneously.
4. Probabilistic decision framework implements weighted soft voting mechanisms to generate interpretable confidence scores for clinical decision support systems.

The remainder of this paper is organized as follows: Section 2 provides a comprehensive review of related work, tracing the evolution from traditional radiomics approaches to advanced deep learning methodologies and identifying critical gaps in ensemble validation frameworks. Section 3 details our methodology, including the KHCC dataset characteristics, preprocessing pipeline, and the ensemble learning framework combining ResNet50 and Swin Transformer architectures through weighted soft voting. Section 4 presents comprehensive results demonstrating the ensemble model's superior performance with rigorous statistical validation using Friedman and Wilcoxon signed-rank tests, followed by detailed comparative analysis with existing literature, clinical implications, and study limitations. Finally, Section 5 concludes with key findings and future research directions for AI-enhanced surveillance in colorectal cancer management.

## 2 Related work

Recent years have witnessed rapid progress in the application of artificial intelligence (AI) for detection and prognosis for colorectal cancer (CRC) liver metastasis detection and prognosis. A diverse body of research has explored radiomics, traditional machine learning, and deep learning approaches across multiple imaging modalities including CT, MRI, and PET/CT, as well as non-imaging clinical biomarkers. While these studies report promising results, they vary widely in methodology, dataset scale, and validation rigor, leading to challenges in reproducibility and clinical translation. The following review critically examines prior work, tracing the evolution of AI methods, comparing performance across modalities, analyzing dataset and validation limitations, and identifying current gaps that motivate our proposed approach.

### 2.1 Evolution of AI approaches

The application of artificial intelligence in predicting liver metastases has evolved significantly, transitioning from traditional machine learning and radiomics to more sophisticated deep learning architectures and novel computational paradigms. As shown in Table 1, early approaches, such as those in Wang J. P. et al. (2025) and Jing et al. (2025), relied on conventional machine learning algorithms like Random Forest and Support Vector Machines (SVM) combined with radiomics feature extraction from CT and MRI scans. These studies established the feasibility of using quantitative image features to predict metachronous liver metastases (MLM). More recent work, like Miyamoto et al. (2025), continues to refine this approach, using Random Forest and Boruta algorithms to predict chemotherapy response from CT radiomics with high accuracy. The field has since progressed toward deep learning, as demonstrated in Lu et al. (2021), Xia et al. (2024), and Kinoshita et al. (2023), which utilize Convolutional Neural Networks (CNNs) and Vision Transformers (ViTs) to automatically learn hierarchical features. This shift reduces the need for manual feature engineering. A key advancement is the focus on model interpretability; Wu et al. (2025) developed a model for stage II CRC using an Artificial Neural Network (ANN) and explained its predictions with the SHAP algorithm, addressing the “black box” problem. The trend toward multimodal integration is also prominent. Li et al. (2025) developed a metabolic-imaging model, combining radiomics with metabolic biomarkers to enhance prognostic prediction. A novel direction is presented in Rocca et al. (2021), which introduced Formal Methods (FMs) as an alternative to traditional AI, demonstrating high precision in very small datasets by using mathematical logic to verify predefined properties rather than learning from large volumes of data. The comprehensive review in Huang et al. (2025) effectively summarizes this evolution, covering the spectrum from conventional ML to advanced deep learning and its applications in precision medicine.

**Table 1:** Comparative summary of recent deep learning approaches for liver metastasis prediction.

**Paper**	**Dataset/patients**	**Imaging modality**	**Method**	**Gap/limitation**	**Contribution**	**Results**
Wang J. P. et al. (2025)	157 CRC pts (retro)	CT & MRI	Radiomics (RF)	Small sample, single center, limited multimodal benefit.	First study to integrate CT and MRI for MLM prediction.	Merged with AUC: 0.82.
Feng et al. (2025)	865 CRC pts (retro/pro)	Clinical Lab Data	ML (RF)	Single-center, cross-sectional design.	Developed a cost-effective model using standard lab data.	CRLM-Lab6 model AUC: 0.94.
Lu et al. (2021)	1,028 mCRC pts (trial)	CT	DL (CNN-RNN)	Single trial data, limited interpretability.	Showed DL utility on serial CTs for early response prediction.	DL-Nomo C-Index: 0.694.
Xia et al. (2024)	206 pts (multi-national)	CT	DL (CNN, ViT)	Excluding non-liver metastases, needs prospective validation.	Developed an automated pipeline mimicking a radiologist's workflow.	RECORD pipeline avg. AUC: 0.981.
Wang J. et al. (2025)	Systematic review	CT, MRI, genetic	DL	Underuse of 3D/dynamic imaging, lack of generalizability.	Provided a comprehensive overview and proposed a benchmark framework.	Reported top accuracy of 99.38% in reviewed studies.
Guo et al. (2024)	574 uCRLM pts (retro)	Clinical Data	ML (RSF)	Retrospective, potential sample size bias.	Externally validated a prognostic model for IAIT-treated patients.	3-year AUC: 0.873 (train), 0.730 (external).
Jing et al. (2025)	51,265 CRC pts (SEER)	Clinical Data	ML (RF)	Lacks some clinical/treatment data.	First large-scale, population-based ML model for metastasis prediction.	RF model AUC: 0.956 (internal), 0.912 (external).
Wu et al. (2024)	212 CRC pts (retro)	CT	Radiomics (RF)	Retrospective, single-center, small sample.	Developed a fusion model integrating radiomics and clinical data.	Fusion model AUC: 0.761.
Kanan et al. (2024)	Meta-analysis (33 studies)	US, CT, MRI	Radiomics & DL	High study heterogeneity, mostly retrospective.	Evaluated AI for recurrence prediction, stressing quality assessment.	Pooled AUC for HCC-SR recurrence: 0.86.
Wang J. P. et al. (2025)	121 pts (retro)	CT	DL Image Recon. (DLIR)	Retrospective, single manufacturer.	Showed advanced reconstruction can improve metastasis detection.	DLIR-high improved metastasis detection.
Huang et al. (2025)	769 Stage II CRC pts (multi-center)	CT	ML (ANN), SHAP	Retrospective, only portal venous phase.	Developed an interpretable model to guide ACT decisions.	Combined model AUC: 0.846 (validation).
Li et al. (2025)	Review article	Multi-modal	AI/ML (SVM, RF, DL)	Data heterogeneity, lack of standardization.	Comprehensive overview of AI's role in CRLM classification.	N/A
Miyamoto et al. (2025)	197 CRLM pts (retro)	CT	ML (ensemble)	Single-center, initial data leakage risk.	Developed a robust model, highlighting and mitigating data leakage.	3-month recurrence model AUC: 0.723.
Kinoshita et al. (2023)	150 CRLM pts (retro)	CT	Radiomics (RF)	Small sample, retrospective, manual segmentation.	Demonstrated utility of ML for predicting chemotherapy response.	Validation AUC: 0.87 for chemo response.
Xia et al. (2024)	543 HCC pts (retro)	CT	DL (DenseNet121)	Single-center, only solitary HCCs.	Developed a DL model to predict early postoperative HCC recurrence.	Test AUC: 0.71 for early recurrence.
Chen et al. (2025)	Meta-analysis (17 studies)	CT, MRI, US	AI (ML/DL)	High study heterogeneity, regional bias.	Systematically evaluated AI's diagnostic accuracy for metastasis.	Pooled AUC: 0.91 for distant metastasis.
Rocca et al. (2021)	30 CRC pts (pilot)	CT	Formal Methods	Very small sample, manual segmentation.	Introduced Formal Methods as a novel approach for small cohorts.	Accuracy: 93.3%, Precision: 100%.

### 2.2 Imaging modality analysis

The choice of imaging modality remains a critical factor, with CT being the most common due to its wide availability and detailed anatomical resolution, as seen in numerous studies including Wang J. P. et al. (2025), Huang et al. (2025), Li et al. (2025), Miyamoto et al. (2025), Kinoshita et al. (2023), and Rocca et al. (2021). Research continues to optimize CT's utility; for example, Kanan et al. (2024) showed that deep learning image reconstruction (DLIR) significantly improves metastasis detection over standard methods. While MRI is noted for its superior soft-tissue contrast, multimodal approaches combining CT and MRI (Wang J. P. et al., 2025) have not yet shown a statistically significant advantage, indicating a need for more sophisticated fusion techniques. The meta-analysis in Chen et al. (2025) provides a high-level comparison, reporting a pooled Area Under the Curve (AUC) of 0.91 across modalities for predicting distant metastasis but also noting that MRI may offer higher specificity. The systematic review in Grover and Gupta (2024) reinforces that while CT and MRI are standard, advanced techniques like 3D and dynamic imaging are still not fully exploited, despite their potential to offer more comprehensive spatial and functional information.

### 2.3 Performance comparison across studies

The performance of AI models varies widely, reflecting the diversity of methods and data. Radiomics-based machine learning models continue to show strong performance, with AUCs for predicting treatment response and recurrence ranging from 0.761 to 0.87 (Jing et al., 2025; Miyamoto et al., 2025). Deep learning models often achieve higher metrics. The RECORD pipeline in Xia et al. (2024) reported an average AUC of 0.981 for response classification, while the interpretable model in liver (11) achieved an AUC up to 0.846 in external validation for predicting recurrence risk. The meta-analysis in Chen et al. (2025) found a robust pooled AUC of 0.91 for predicting distant metastasis. Even models focused on more challenging tasks, such as the 3-month recurrence prediction in Li et al. (2025), reached a clinically useful AUC of 0.723. A novel approach using Formal Methods in Rocca et al. (2021) reported 100% precision on a small pilot cohort, suggesting its potential where large datasets are unavailable. In contrast, models based solely on clinical data also demonstrate strong predictive power; Feng et al. (2025) achieved an AUC of 0.94 using only lab tests, and Guo et al. (2024) reached an AUC of 0.956 on the large SEER database, highlighting that data scale can be as important as data type.

### 2.4 Dataset scale and validation challenges

The scale of datasets and the rigor of validation methods are crucial for the clinical translation of AI models. Many studies are still limited to smaller, single-center retrospective cohorts (Wang J. P. et al., 2025; Jing et al., 2025; Li et al., 2025; Miyamoto et al., 2025), which may not be generalizable. The pilot studied in Rocca et al. (2021) intentionally used a small cohort (30 patients) to demonstrate the utility of Formal Methods in such scenarios. In contrast, large-scale, multi-center studies are becoming more common. (Lu et al., 2021) utilized a cohort of 769 patients from three medical centers, while Lu et al. (2021) and Xia et al. (2024) leveraged data from international clinical trials. The most extensive dataset was used in Guo et al. (2024), which included over 50,000 patients from the SEER database. These larger studies provide more robust and generalizable models. A critical methodological challenge highlighted by Li et al. (2025) is the risk of “data leakage,” where post-operative information inadvertently contaminates the training of a pre-operative prediction model, leading to artificially inflated performance metrics. This underscores the need for stringent temporal validation. The meta-analyses in Wu et al. (2024) and Chen et al. (2025) both confirm that most studies are retrospective and often geographically concentrated, emphasizing the persistent need for prospective and diverse external validation.

### 2.5 Current gaps and limitations

Despite significant progress, several key challenges persist. The lack of methodological standardization across studies, from imaging protocols to model development, remains a major barrier to comparing and integrating findings, a limit noted in the review's Wu et al. (2024) and Huang et al. (2025). The “black box” nature of complex models like deep neural networks is another significant hurdle for clinical adoption. In response, recent work has focused on interpretability, with (Wu et al., 2025) successfully using the SHAP algorithm to explain feature contributions in their predictive model, making the results more transparent to clinicians. Data availability and quality continue to be a primary constraint. As highlighted in Li et al. (2025), a major and often overlooked limitation is the risk of data leakage in prognostic studies, which can render models clinically invalid. This calls for more rigorous study design and peer review. The challenge of working with small datasets is addressed by Rocca et al. (2021), which proposes Formal Methods as a viable alternative to data-hungry deep learning models. Finally, the meta-analysis in Chen et al. (2025) and review in Huang et al. (2025) reiterate a common theme: the vast majority of studies are retrospective and lack robust, prospective external validation. Bridging this gap between model development and clinical utility is the most critical step for the future of AI in oncology.

In summary, the literature demonstrates substantial progress from handcrafted ML to transformer-augmented DL approaches, with robust performances achieved in large multicenter cohorts. Nevertheless, generalizability, reproducibility, and clinical integration remain critical barriers. Furthermore, the paradoxical strength of clinical-only models suggests that future work must focus on holistic, multimodal integration rather than siloed imaging analysis. These gaps collectively motivate the present work, which seeks to develop a clinically translatable AI framework for early and accurate detection of CRC liver metastases.

## 3 Methodology

**Figure 1:**
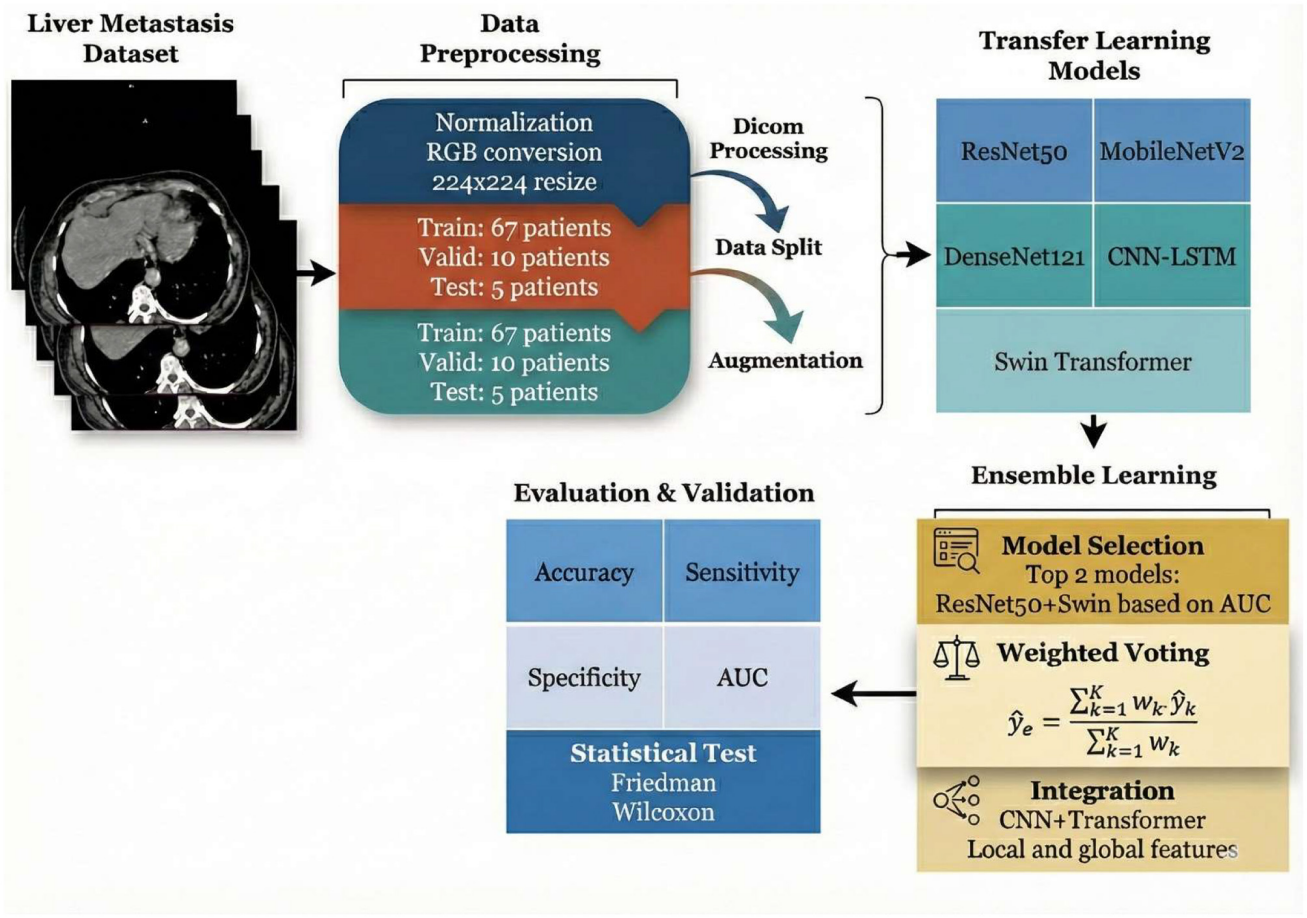
Framework of the deep learning-based liver metastasis detection system employing ensemble learning and comprehensive performance evaluation.

This study employed a comprehensive machine learning pipeline for liver metastasis detection and classification using medical imaging data. The methodology in Figure 1 began with a curated dataset of liver CT scans from 82 patients, which underwent systematic preprocessing including DICOM format handling, image normalization, RGB conversion, and standardization to 224 × 224-pixel resolution. The dataset was strategically partitioned into training (67 patients), validation (10 patients), and test (5 patients) sets, with data augmentation techniques applied to enhance model robustness and generalization. Five state-of-the-art deep learning architectures were evaluated as transfer learning models: ResNet50, MobileNetV2, DenseNet121, CNN-LSTM, and Swin Transformer. To optimize predictive performance, an ensemble learning approach was implemented, featuring model selection based on Area Under the Curve (AUC) metrics, with the top-performing models (ResNet50 and Swin Transformer) combined through weighted voting mechanisms. The integration strategy leveraged both convolutional neural networks and transformer architectures to capture local and global image features effectively. Model performance was rigorously assessed using multiple evaluation metrics including accuracy, sensitivity, specificity, and AUC, with statistical validation performed through Friedman and Wilcoxon tests to ensure the reliability and significance of the results.

### 3.1 Dataset

**Figure 2:**
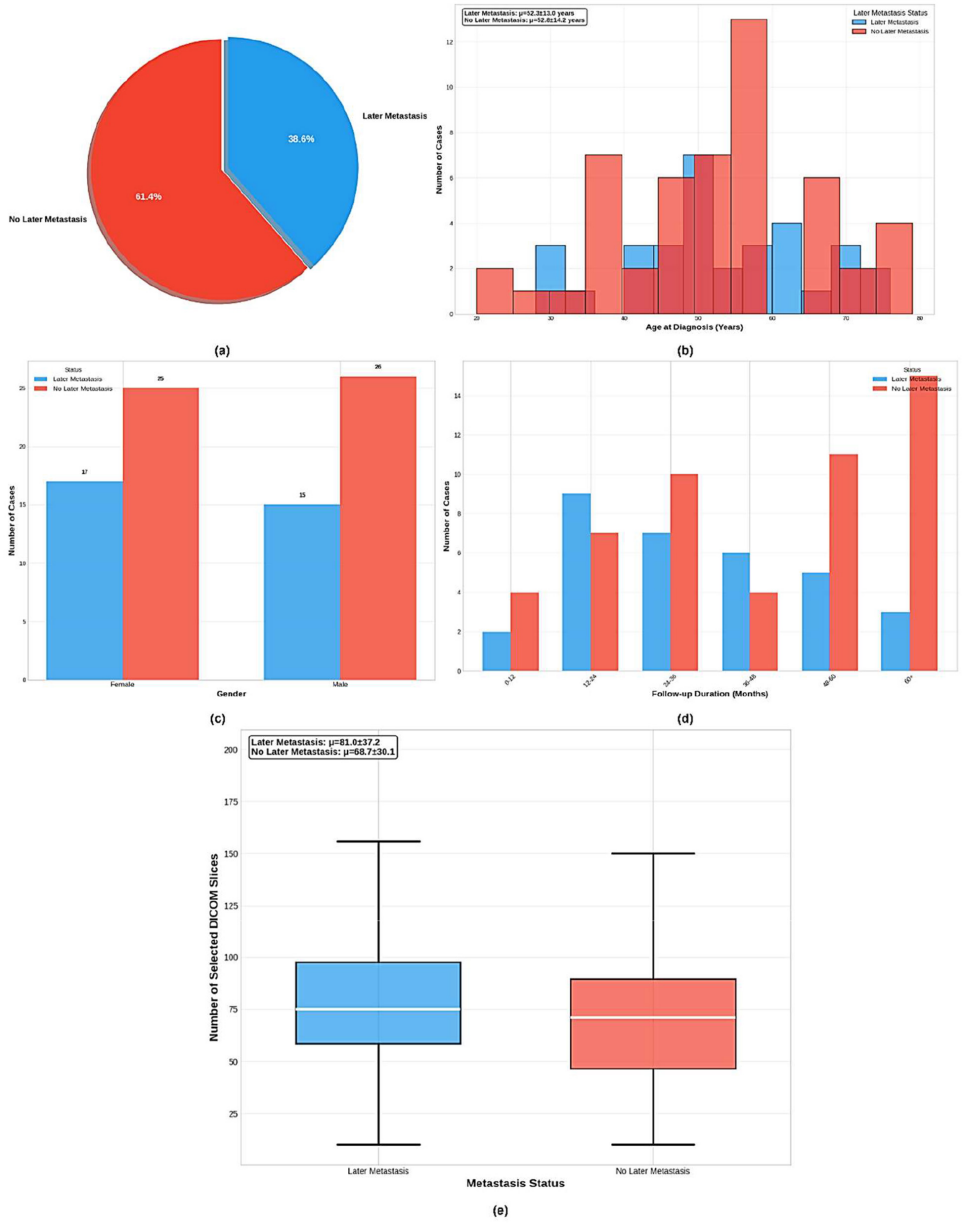
Comprehensive analysis of the KHCC liver metastasis cohort (*n* = 83) showing: **(a)** distribution of later metastasis cases (38.6% vs. 61.4%), **(b)** age demographics by metastasis status, **(c)** gender distribution across groups, **(d)** follow-up duration patterns, and **(e)** DICOM slice selection analysis for radiological image curation quality assessment.

**Figure 3:**
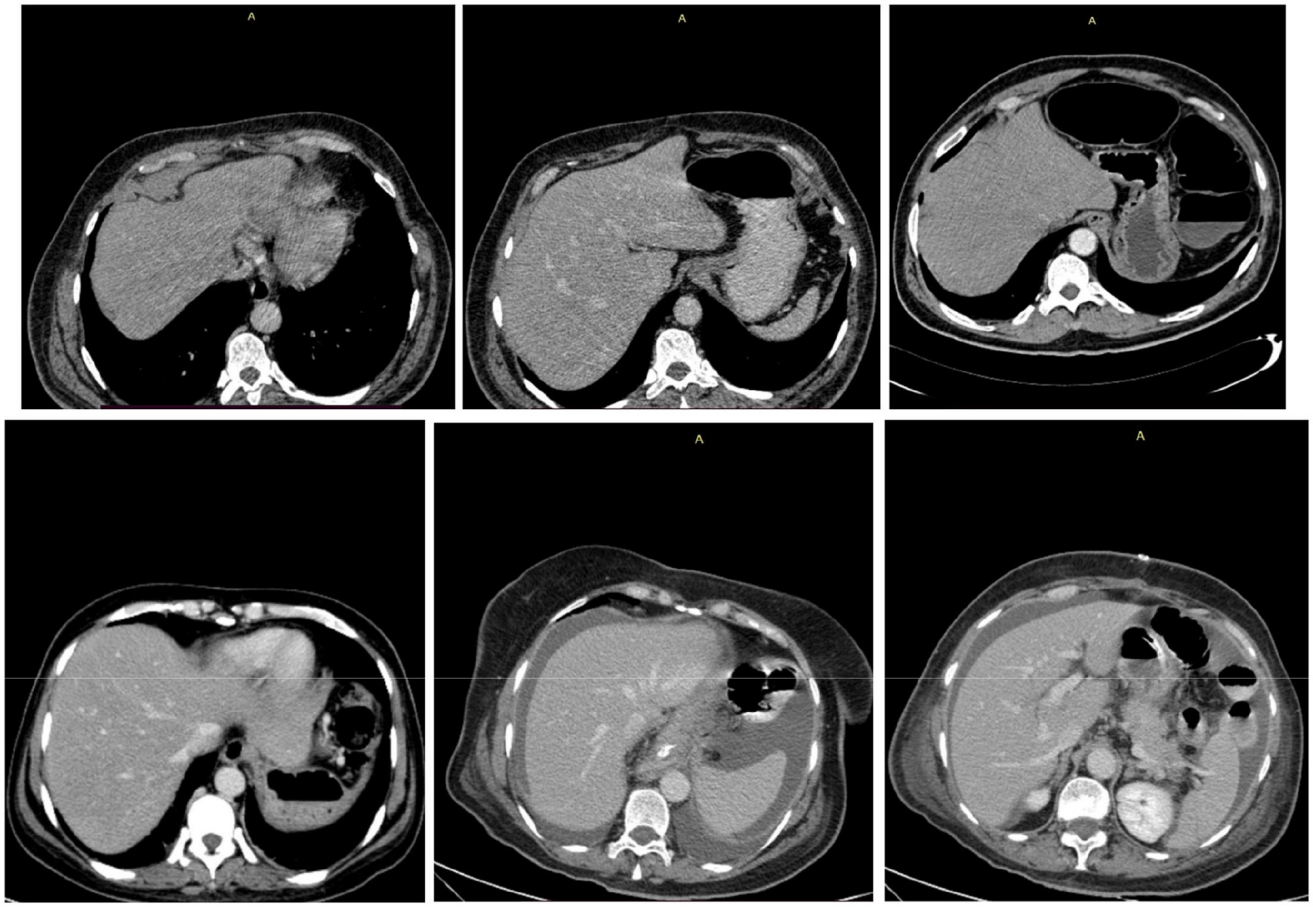
Sample of liver metastasis CT images.

Based on the dataset information shown in Figure 2, the King Hussein Cancer Center (KHCC) Liver Metastasis Predictive Analysis Study represents a comprehensive clinical dataset for advanced medical imaging and clinical outcomes research. The dataset comprises 83 total cases collected during the study period from 2019 to 2024 at KHCC, with 32 cases (38.6%) presenting with later metastasis and 51 cases (61.4%) showing no later metastasis development. The cohort demonstrates balanced gender distribution with slight female predominance in the later metastasis group. Age distribution analysis reveals that patients with later metastasis had a mean age of 54.8 ± 13.6 years, while those without later metastasis averaged 54.4 ± 14.9 years, indicating comparable age demographics across groups. Follow-up periods extended to 34.4 ± 23.0 months for the metastasis group and 47.1 ± 26.1 months for the non-metastasis group. The dataset maintains complete clinical validation with 100% data completeness across all critical variables, including clinical validation, radiological assessment, DICOM image quality, treatment history, metastasis status, gender information, age at diagnosis, and patient demographics. Professional DICOM processing involved advanced medical imaging preprocessing with noise reduction and standardization, along with automated quality control through manual validation by certified radiologists as shown in Figure 3, ensuring IRB-compliant data anonymization and privacy protection protocols throughout the research process. This study was approved by the KHCC Institutional Review Board (IRB# 24KHCC235F) with appropriate ethical oversight for retrospective medical imaging research.

### 3.2 Preprocessing

The dataset preprocessing pipeline implemented a multi-stage approach to prepare DICOM medical images for deep learning classification. Initially, 82 patients with liver DICOM images were identified, comprising 50 patients without metastasis (class 0) and 32 patients with later metastasis (class 1) as shown in Table 2. The patient cohort was strategically split at the patient level to prevent data leakage, with 67 patients allocated for training, 10 patients for validation, and 5 patients for testing. Each patient contributed multiple DICOM slices, resulting in varying numbers of images per split. The DICOM preprocessing involved reading pixel arrays with forced loading, normalizing intensity values to the 0–255 range using min-max scaling, and converting grayscale images to 3-channel RGB format for compatibility with pretrained models. To address the inherent class imbalance in the training set, a cost-sensitive learning strategy was adopted instead of oversampling. Specifically, a weighted Binary Cross-Entropy (BCE) loss function was implemented, where a calculated positive class weight (*Pos*_*weight*_) was applied to the minority class (metastasis) based on the ratio of negative to positive samples (Buda et al., 2018). This approach inherently penalizes misclassifications of the minority class more heavily during backpropagation, effectively balancing the learning process without introducing data redundancy or the potential leakage risks associated with replicating images. Image preprocessing included resizing to 224 × 224 pixels, tensor conversion, and ImageNet normalization (mean = [0.485, 0.456, 0.406], std = [0.229, 0.224, 0.225]). This strategy ensures robust model optimization while maintaining the integrity of the natural clinical data distribution.

**Table 2:** Patient and image distribution across dataset splits with class balancing.

**Split**	**Total patients**	**Non metastasis**	**metastasis**	**Total images**	**Non metastasis images**	**Metastasis images**	**Class ratio (1:0)**
Training	67	41	26	5,271	3,355	1,916	0.57
Validation	10	6	4	527	331	196	0.59
Test	5	3	2	416	273	143	0.52

### 3.3 Overview of the proposed approach

This study proposes a comprehensive deep learning framework for liver metastasis prediction using the Liver Metastasis Prediction Dataset. The methodology employs six state-of-the-art pretrained convolutional neural networks (CNNs) and transformer architectures as feature extractors through transfer learning, followed by ensemble learning to combine the top-performing models. The framework integrates diverse architectural paradigms to leverage complementary feature representations for robust metastasis classification.

#### 3.3.1 ResNet50

ResNet50V2 implements the residual learning framework proposed by He et al. (2016) to address the degradation problem in deep networks. Unlike the original V1, the V2 variant utilizes pre-activation residual units where Batch Normalization (BN) and ReLU activation precede the convolution operations. This improves gradient propagation through the network. The output *y*_*l*_of the *l*-th residual block is formally defined as:


$$\begin{array}{l} y_{l}=h(x_{l})+F(x_{l},\{W_{l}\}) \end{array}$$ (1)

Where *x*_*l*_is the input feature vector, *h*(*x*_*l*_)denotes the identity mapping (skip connection), and Frepresents the residual mapping function to be learned, composed of a stack of 1 × 1, 3 × 3, and 1 × 1 convolutions.

#### 3.3.2 MobileNetV2

MobileNetV2, introduced by Sandler et al. (2018), utilizes inverted residual blocks with linear bottlenecks to optimize computational efficiency while maintaining representational capacity. Unlike standard residual blocks, it expands the input to a higher dimension, applies depthwise convolution, and projects it back to a lower dimension. The operation for an input tensor *x* is formulated as:


$$\begin{array}{l} y={\text{Con}{\text{v}}_{1\times 1}(\text{DWConv}(\text{Conv}}_{1\times 1}\left(x,t)\right)) \end{array}$$ (2)

Where *Conv*_1 × 1_ represents pointwise convolution, DWConv denotes depthwise convolution, and *t* is the expansion factor. The linear bottleneck design removes the non-linear activation at the final output layer to preserve information flow in low-dimensional manifolds.

#### 3.3.3 DenseNet121

DenseNet121 implements dense connectivity patterns as proposed by Huang et al. (2017), where each layer receives feature maps from all preceding layers. The dense block connectivity is defined as shown in Equation 3:


$$\begin{array}{l} x_{l}=H_{l}\left(\left[x_{0},x_{1},\cdot ,\cdots x_{l-1}\right]\right) \end{array}$$ (3)

Where [*x*_0_, *x*_1_, ·, ⋯*x*_*l*−1_] represents the concatenation of feature maps from layers *0,…,l-1*, and *H*_1_(.) denotes the composite function consisting of batch normalization, ReLU activation, and 3 × 3 convolution. This architecture promotes feature reuse and alleviates the vanishing gradient problem.

#### 3.3.4 CNN_LSTM

The CNN-LSTM model represents a hybrid deep learning approach that combines convolutional neural networks with recurrent neural networks for enhanced feature learning and temporal modeling. The architecture employs a CNN backbone as the feature extractor, with the final classification layer replaced by an identity function to output high-dimensional feature vectors. These CNN-derived features are then projected through a linear layer to 128 dimensions before being fed into a bidirectional LSTM network consisting of 2 layers with 128 hidden units each. The bidirectional LSTM processes the projected features as a sequence (treating each image as a single time step), enabling the model to capture both forward and backward temporal dependencies, resulting in 256-dimensional output features (128 × 2 for bidirectional processing). A regularized classifier head with dropout layers (0.7 dropout rate), batch normalization, and ReLU activations processes the LSTM output through two fully connected layers (256 → 64 → 1) before sigmoid activation for binary classification. This hybrid architecture aims to leverage both the spatial feature extraction capabilities of CNNs and the sequential modeling strengths of LSTMs, potentially capturing complex patterns that might be missed by purely convolutional approaches in medical image analysis tasks (Donahue et al., 2015).

#### 3.3.5 Swin transformer

The Swin Transformer, introduced by Liu et al. (2021), implements hierarchical vision transformers using shifted window-based self-attention. Recent advances in transformer-based architecture have demonstrated superior performance over traditional CNNs in complex visual recognition tasks, with attention mechanisms proving particularly effective in drawing connections among different parts of images while maintaining computational efficiency through patch-based processing strategies. The hierarchical attention approach aligns with findings from multi-scale analysis frameworks, where capturing features at different resolution levels and spatial relationships has shown significant improvements in classification accuracy compared to fixed-scale processing methods (Tuncer et al., 2022; Eralp and Sefer, 2024). Transformer-based modules are increasingly integrated into medical image segmentation to capture long-range dependencies and global contextual information, overcoming the locality limitations of traditional Convolutional Neural Networks (CNNs). By leveraging self-attention mechanisms, these architectures effectively model global anatomical relationships while preserving local feature details, resulting in improved segmentation accuracy for complex, irregular structures.

The shifted window multi-head self-attention (SW-MSA) mechanism is computed as shown in Equation 4:


$$\begin{array}{l} A\left(Q,K,V\right)=SoftMax\left(\frac{Q^{T}K}{\sqrt{d}}+B\right)V \end{array}$$ (4)

Where *B*∈*R*^*M*^^2^×*M*^2^ represents the relative position bias matrix, and *d* denotes the query/key dimension. The shifting operation enables cross-window connections while maintaining linear computational complexity with respect to image size.

### 3.4 Transfer learning strategy

All backbone models are initialized with ImageNet pretrained weights to leverage low-level feature representations learned from natural images. We employ a two-stage training strategy. Initially, all pretrained layers are frozen, and only the classifier head undergoes training for 10 epochs. Subsequently, the entire network is fine-tuned with a reduced learning rate of 0.0001 to adapt pretrained features to the liver metastasis domain. This approach has proven highly effective in medical diagnostics, significantly enhancing classification accuracy and convergence speed by fine-tuning established weights for specific tasks such as lesion segmentation or pneumonia detection (Thapar et al., 2025; Khan et al., 2023b).

The feature vector *f*, extracted by the backbone (after Global Average Pooling), is passed through a Dropout layer and a Fully Connected (FC) layer. The probability of malignancy ŷis computed using the Sigmoid activation function, as shown in Equation 5:


$$\begin{array}{l} P\left(y=1|x\right)=\sigma \left(W^{T}f+b\right)=\frac{1}{1+e^{-\left(W^{T}f+b\right)}} \end{array}$$ (5)

Where *W*and *b*are the learnable weights and bias of the final dense layer, and the output represents the probability of the positive class (Metastasis).

### 3.5 Loss functions

The selection of appropriate loss functions is critical for optimizing deep learning models in medical image classification tasks, particularly when dealing with imbalanced datasets common in clinical applications. Our framework implements multiple loss function strategies to ensure robust training and optimal convergence across different class distributions and model architectures.

#### 3.5.1 Cross-entropy loss

The standard cross-entropy loss function is employed as the primary optimization objective as shown in Equation 6:


$$\begin{array}{l} L_{CE}=\frac{1}{N}\overset{N}{\underset{i=1}{\sum }}\overset{C}{\underset{c=1}{\sum }}y_{i,c}\log\left({ŷ}_{i,c}\right) \end{array}$$ (6)

Where *N* denotes the batch size, *y*_*i, c*_ represents the ground truth label, and ŷ_*i, c*_ denotes the predicted probability (Mao et al., 2023).

#### 3.5.2 Focal loss

To address potential class imbalance in the liver metastasis dataset, we additionally implement focal loss as proposed by Thapar et al. (2025) as shown in Equation 7:


$$\begin{array}{l} L_{FL}=\alpha_{c}{\left(1-{ŷ}_{i,c}\right)}^{\gamma }\log\left({ŷ}_{i,c}\right) \end{array}$$ (7)

Where α_*c*_ represents the class-specific weighting factor and γ denotes the focusing parameter (Lin et al., 2017). This formulation down-weights well-classified examples and focuses learning on hard cases.

### 3.6 Model evaluation and selection

All six backbone models undergo identical training procedures using 5-fold cross-validation. Model performance is assessed using multiple metrics including accuracy, precision, recall, F1-score, Macro-F1, and area under the ROC curve (AUC). The AUC serves as the primary selection criterion due to its robustness to class imbalance and its ability to evaluate model performance across all classification thresholds as shown in Equation 8:


$$\begin{array}{l} AUC=\int_{0}^{1}TPR\left(t\right)d\left[FPR\left(t\right)\right] \end{array}$$ (8)

Where *TPR* denotes the true positive rate and *FPR* represents the false positive rate at threshold *t*. The AUC metric provides a single scalar value summarizing the model's discriminative ability across all possible decision thresholds.

The top two performing models based on validation AUC scores are selected for ensemble learning.

### 3.7 Ensemble learning methodology (weighted soft voting)

The ensemble combines predictions from the top-performing models using a Weighted Soft Voting mechanism. Unlike hard voting, which relies on discrete labels, soft voting aggregates the predicted probabilities. Let *P*_*k*_(*y* = 1∣*x*) be the probability output of the *k*-th model. The weight *w*_*k*_for each model is dynamically assigned based on its validation AUC score to prioritize more robust models as shown in Equation 9:


$$\begin{array}{l} w_{k}=\frac{{\text{AUC}}_{k}}{\sum_{j=1}^{K}{\text{AUC}}_{j}} \end{array}$$ (9)

The final ensemble probability ${\hat{P}}_{ensemble}\;$ is derived from the weighted linear combination of individual probabilities as shown in Equation 10:


$$\begin{array}{l} {\hat{P}}_{ensemble}\left(y=1|x\right)=\overset{K}{\underset{k=1}{\sum }}w_{k}\cdot P_{k}\left(y=1|x\right) \end{array}$$ (10)

Where *K* represents the number of selected models. The final class label is determined by applying a threshold of 0.5 to ${\hat{P}}_{ensemble}$.

### 3.8 Rationale for CNN-transformer ensemble

The combination of CNN-based architecture with transformer-based models leverages complementary inductive biases for improved generalization. CNNs excel at capturing local spatial patterns and hierarchical feature representations through convolution operations and pooling mechanisms. Conversely, transformers capture long-range dependencies and global contextual information through self-attention mechanisms. For liver metastasis prediction, this synergy enables the model to simultaneously detect local pathological patterns (via CNNs) and global anatomical relationships (via transformers). The ensemble approach mitigates individual model limitations while enhancing robustness to variations in imaging conditions and pathological presentations. To aggregate these complementary features, a soft voting strategy was strictly employed over hard voting. Hard voting was excluded because it relies on discrete class labels (majority rule), which create undefined tie scenarios in a two-model ensemble. Furthermore, hard voting discards valuable probabilistic confidence scores. By using soft voting, the ensemble preserves the continuous probability outputs, ensuring that the final prediction reflects not just the class label, but the combined confidence of both architectures, resulting in a more discriminative and calibrated diagnostic outcome.

### 3.9 Performance evaluation methods

Accuracy, precision, sensitivity, specificity, F1 score, and AUC as shown in Equations 11–19 are the metrics used to assess the performance of our multi-model approach for liver metastasis detection. Each metric provides valuable insight into the model's diagnostic capabilities, and their comprehensive evaluation ensures robust assessment of the model's clinical utility for liver metastasis prediction.

- TP: True Positives (cases with liver metastasis correctly identified)
- TN: True Negatives (correctly identified non-metastatic cases)
- FP: False Positives (non-metastatic cases incorrectly classified as metastatic)
- FN: False Negatives (metastatic cases misclassified as non-metastatic)
- Accuracy

Measures the proportion of all correct predictions (both true positives and true negatives) out of the total number of cases.


$$\begin{array}{l} Accuracy=\frac{TP+TN}{TP+TN+FP+FN} \end{array}$$ (11)

- Precision

Or called (Positive Predictive Value): Quantifies the accuracy of the positive predictions, representing the fraction of predicted metastatic cases that are metastatic.


$$\begin{array}{l} \text{Precision}=\frac{TP}{TP+FP} \end{array}$$ (12)

- Sensitivity (true positive rate)

Or called recall, it measures the model's ability to correctly identify all actual metastatic cases.


$$\begin{array}{l} \text{Sensitivity }=\frac{TP}{TP+FN} \end{array}$$ (13)

- Specificity (true negative rate)

Measures the model's ability to correctly identify all actual non-metastatic cases.


$$\begin{array}{l} \text{Specificity }=\frac{TN}{TN+FP} \end{array}$$ (14)

- F1 Score

The harmonic mean of precision and sensitivity provides a balanced measure of performance, especially important for imbalanced medical datasets.


$$\begin{array}{l} \text{F}1\text{ Score}=2^{*}\frac{\text{Precisio}{\text{n}}^{*}\text{Sensitivity }}{\text{Precision}+\text{Sensitivity}} \end{array}$$ (15)

- Area Under the Curve (AUC)

Represents the model's ability to distinguish between positive and negative classes across all classification thresholds. An AUC of 1.0 indicates a perfect classifier.


$$\begin{array}{l} \text{TPR}=\frac{\text{TP }}{\text{TP}+\text{FN}} \end{array}$$ (16)

$$\begin{array}{l} \text{FPR}=\frac{\text{FP }}{\text{FP}+\text{TN}} \end{array}$$ (17)

$$\begin{array}{l} AUC=\int_{0}^{1}TPRd\left(FPR\right) \end{array}$$ (18)

$$\begin{array}{l} AUC=\overset{n-1}{\underset{i=1}{\sum }}{\left(FPR_{i+1}-FPR_{i}\right)}^{*}\frac{FPR_{i}+FPR_{i+1}}{2} \end{array}$$ (19)

The Friedman test as shown in Equation 20 is employed for statistical comparison of multiple algorithms across different datasets.


$$\begin{array}{l} \text{Friedman}=x_{F}^{2}=\frac{12N}{k\left(K+1\right)}\sum_{j=1}^{k}{\left({\bar{R}}_{j}-\frac{k+1}{2}\right)}^{2} \end{array}$$ (20)

Where $x_{F}^{2}$ represents the test statistic derived from the discrepancies between the average ranks and the anticipated rank $\frac{k+1}{2}$ in the Friedman test; in this context, *N* signifies the number of datasets, *k* indicates the total number of algorithms, and ${\bar{R}}_{j}$ representing its average rank.

- Wilcoxon Signed-Rank Test

The Wilcoxon signed-rank test as shown in Equation 21 is used for pairwise *post-hoc* comparisons following the Friedman test to determine which specific algorithms differ significantly.


$$\begin{array}{l} w=\overset{n}{\underset{i=1}{\sum }}{\sqrt{\left(x_{i}-y_{i}\right)}}^{*}R_{i} \end{array}$$ (21)

Where *W* represents the test statistic, *x*_*i*_ and *y*_*i*_ are paired observations from two algorithms, and *R*_*i*_ is the rank of the absolute difference *|x*_*i*_ *- y*_*i*_*|*.

This study demonstrates the effectiveness of using ensemble deep learning models for liver metastasis classification. By combining multiple architectures and employing rigorous statistical evaluation through Friedman and Wilcoxon tests, we show significant improvements in classification performance. Comprehensive evaluation metrics confirm the robustness of the ensemble approach, providing a reliable and statistically validated solution for real-world liver metastasis detection in medical imaging.

## 4 Results and discussion

All experiments were conducted on a high-performance computing system equipped with dual NVIDIA GeForce RTX 4090 GPUs, each providing 24 GB of VRAM for a total of 48 GB GPU memory. This substantial computational capacity enabled efficient training of large-scale deep learning models and facilitated batch processing of the extensive DICOM image dataset. The parallel GPU configuration significantly reduced training time while allowing for larger batch sizes and more complex model architectures to be explored effectively. This section presents the comprehensive results of our deep learning ensemble models, including implementation details and hyperparameter configurations, results analysis, statistical validation using Friedman and Wilcoxon tests, comparative analysis with radiomics and advanced deep learning methodologies, clinical impact and translation potential, and key limitations of the study.

### 4.1 Implementation details and hyperparameter selection

The models were implemented using the PyTorch framework and trained on an NVIDIA GPU. The selection of hyperparameters was a critical step guided by a combination of common practices in medical image analysis, values reported in the source literature for the pre-trained models, and empirical tuning based on performance on our dedicated validation set.

All models were trained using the AdamW optimizer, chosen for its effective weight decay implementation, and a Binary Cross-Entropy (BCELoss) function. To prevent overfitting, we employed several regularization techniques:

1. Heavy dropout layers (p=0.7) in the classifier head.
2. An early stopping mechanism that monitored the validation loss, terminating the training if no improvement was observed for 6 consecutive epochs.
3. A learning rate scheduler (ReduceLROnPlateau) which reduced the learning rate by a factor of 0.7 if the validation loss plateaued for 3 epochs.

The final set of hyperparameters used for training all models is detailed in Table 3.

**Table 3:** Hyperparameters used for model training and selection.

**Hyperparameter**	**Value**	**Rationale/selection method**
Image size	224 × 224	Standard input size for most pre-trained models (e.g., ResNet, DenseNet, Swin).
Batch size	6	Determined empirically as the maximum size that fit within GPU memory constraints while ensuring stable training.
Pre-training	True	Utilized ImageNet pre-trained weights to leverage transfer learning, which is standard for medical imaging tasks.
Dropout rate (Classifier)	0.7	A relatively high value selected after preliminary experiments to provide strong regularization and combat overfitting on the dataset.
LSTM hidden units	128	(For CNN-LSTM) Chosen as a balanced value to capture sequential information without excessive complexity.
LSTM layers	2	(For CNN-LSTM) Selected to allow for more complex temporal feature extraction.
Optimizer	AdamW	Chosen over standard Adam for its improved weight decay implementation, which often yields better generalization.
Initial learning rate	5e-4 (0.0005)	Selected based on empirical tuning; this value provided a good balance between convergence speed and stability.
Weight decay	1e-4 (0.0001)	A common default value for AdamW that provides light $L_2$ regularization.
Max epochs	25	Set as an upper limit. All models stopped earlier due to the early stopping criterion.
Loss function	Binary Cross-Entropy (BCELoss)	Standard loss function for binary classification problems.
Early stopping patience	6 epochs	Allowed the model to continue for 6 epochs without validation loss improvement before stopping.
Gradient clipping norm	1.0	Applied to prevent exploding gradients, ensuring training stability.
LR scheduler	ReduceLROnPlateau	Chosen to dynamically decrease the learning rate when the validation loss plateaus.
LR scheduler factor	0.7	A moderate reduction factor, allowing for finer-grained tuning as training progresses.
LR scheduler patience	3 epochs	Reduced the learning rate if validation loss did not improve for 3 consecutive epochs.

### 4.2 Results analysis

**Figure 4:**
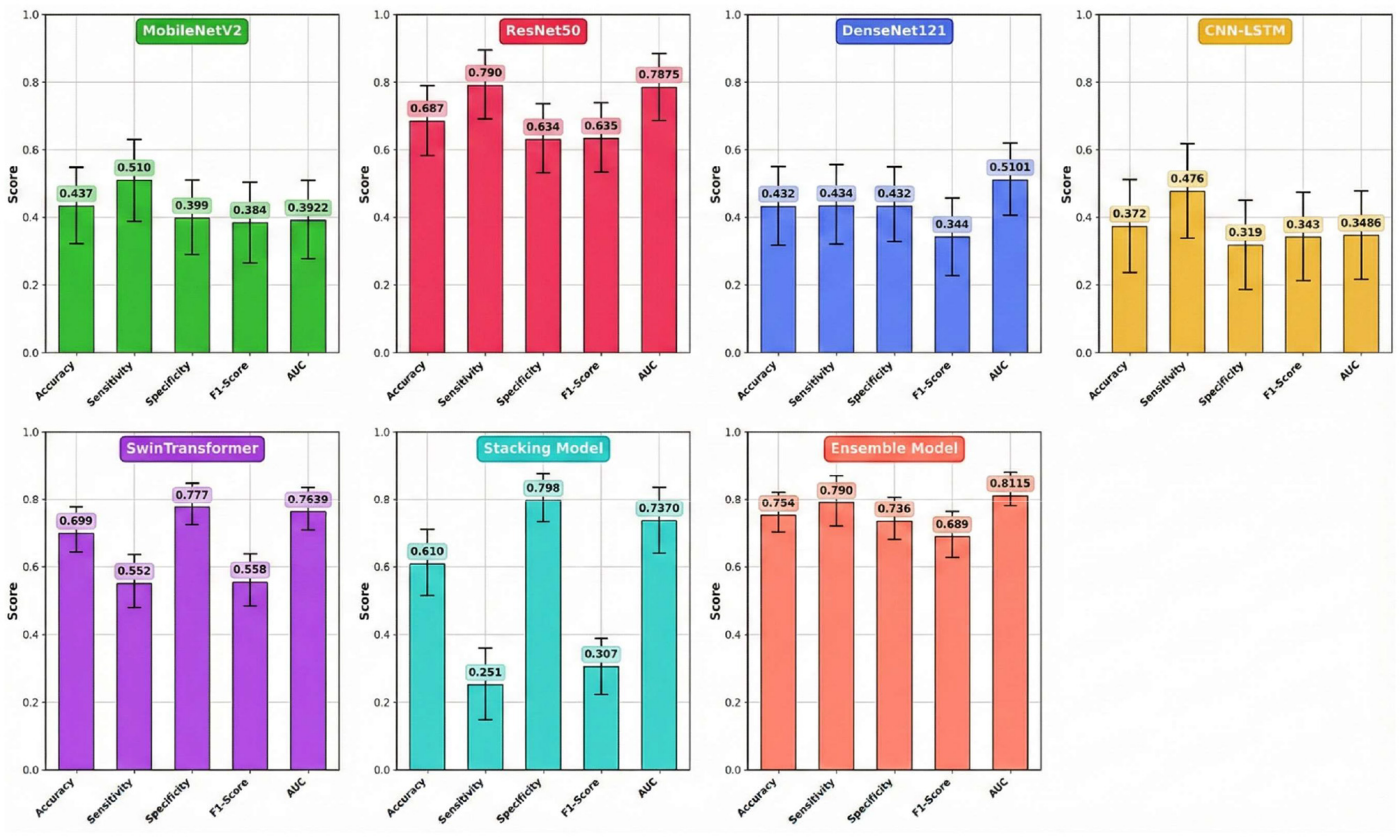
Comparative performance analysis of individual and ensemble models.

The comprehensive performance of each model on the independent test set, calculated as the average of five experimental runs to ensure statistical reliability, is detailed in Table 4 and visually summarized in Figure 4. The results clearly demonstrate the superior efficacy and stability of the multi-model Ensemble approach for this complex medical imaging task. The Ensemble model emerged as the top performer, achieving a robust mean accuracy of 0.754 (±0.039) and an Area Under the Curve (AUC) of 0.8115 (±0.035). The relatively low standard deviation values indicate that the Ensemble model offers consistent predictions across different data folds, minimizing the variability often seen in individual deep learning models. Furthermore, it maintained a well-balanced profile with a sensitivity of 0.790 (±0.045) and a specificity of 0.736 (±0.041), making it a reliable diagnostic aid for identifying both positive and negative cases.

**Table 4:** The performance metrics for each model evaluated on the independent test set (Mean ± STD).

**Model**	**Accuracy**	**Sensitivity**	**Specificity**	**F1-Score**	**AUC**	**Training time (min)**
MobileNetV2	0.437 ± 0.071	0.510 ± 0.082	0.399 ± 0.065	0.384 ± 0.073	0.3922 ± 0.062	33.8
ResNet50	0.687 ± 0.058	0.790 ± 0.069	0.634 ± 0.061	0.635 ± 0.059	0.7875 ± 0.051	32.9
DenseNet121	0.432 ± 0.068	0.434 ± 0.075	0.432 ± 0.070	0.344 ± 0.069	0.5101 ± 0.065	34.0
CNN-LSTM	0.372 ± 0.085	0.476 ± 0.091	0.319 ± 0.088	0.343 ± 0.082	0.3486 ± 0.079	33.0
Swintransformer	0.699 ± 0.051	0.552 ± 0.062	0.777 ± 0.048	0.558 ± 0.055	0.7639 ± 0.042	41.8
Stacking model	0.610 ± 0.072	0.251 ± 0.095	0.798 ± 0.059	0.307 ± 0.085	0.7370 ± 0.068	>1
Ensemble model	0.754 ± 0.039	0.790 ± 0.045	0.736 ± 0.041	0.689 ± 0.043	0.8115 ± 0.035	>1

While the Stacking model did not match the Ensemble's overall accuracy (0.610 ± 0.072), it notably achieved the highest specificity among all models at 0.798 (±0.059). However, this strength was heavily offset by a critically low mean sensitivity of 0.251 (±0.095), rendering it less effective for screening purposes where missing positive cases is a primary concern. Among the individual architectures, Swin Transformer and ResNet50 proved to be the most effective standalone models, yielding mean accuracies of 0.699 (±0.051) and 0.687 (±0.058), respectively. Interestingly, ResNet50 matched the Ensemble model's sensitivity (0.790), suggesting it is particularly capable of detecting metastatic features, though with slightly less overall precision. Conversely, MobileNetV2, DenseNet121, and the hybrid CNN-LSTM models failed to generalize well on this dataset. Their mean AUC values ranged between 0.3486 and 0.5101, often falling near or below the threshold of random chance (0.5), which confirms their unsuitability for this specific predictive application. However, the extended training duration for the Swin Transformer is attributed to the computational complexity of its self-attention mechanisms compared to CNN-based architectures. At the same time, the negligible times for the Ensemble and Stacking models highlight their efficiency in leveraging pre-trained base models without requiring significant additional computational resources.

**Figure 5:**
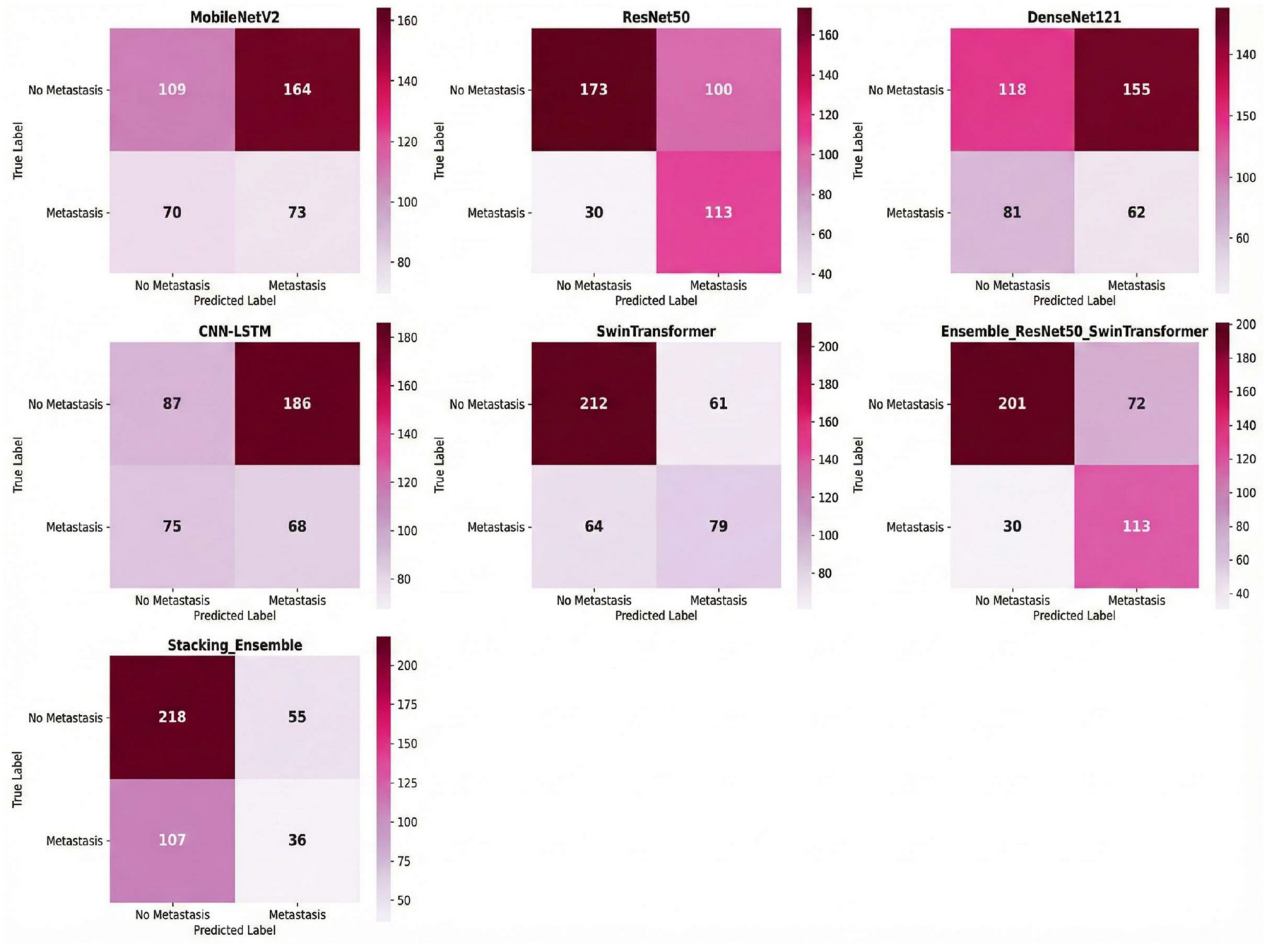
Confusion matrices for model performance on the test set.

Combined ResNet50 and Swin Transformer, demonstrated the most balanced performance with 113 true positives and 201 true negatives, against 72 false positives and 30 false negatives as shown in Figure 5. An analysis of the component models reveals the source of the ensemble's effectiveness: ResNet50 was highly sensitive (TP = 113, FN = 30) but produced many false alarms (FP = 100), whereas the Swin Transformer exhibited the opposite characteristic with high specificity (TN = 212, FP = 61) but lower sensitivity, missing 64 positive cases. By combining these complementary strengths, the ensemble model achieved a more reliable balance.

The stacking model, employing a meta-learner approach with ResNet50 and Swin Transformer as base models, displayed a distinctly different classification pattern with 218 true negatives and only 36 true positives, accompanied by 55 false positives and 107 false negatives. This configuration reflects the stacking model's prioritization of specificity over sensitivity; it excelled at correctly identifying negative cases but failed to capture a substantial portion of positive cases. The high number of false negatives (107) indicates that the meta-learner was overly conservative in its positive predictions, resulting in a lower recall that would be problematic in a clinical setting where missing positive cases carries significant diagnostic consequences.

**Figure 6:**
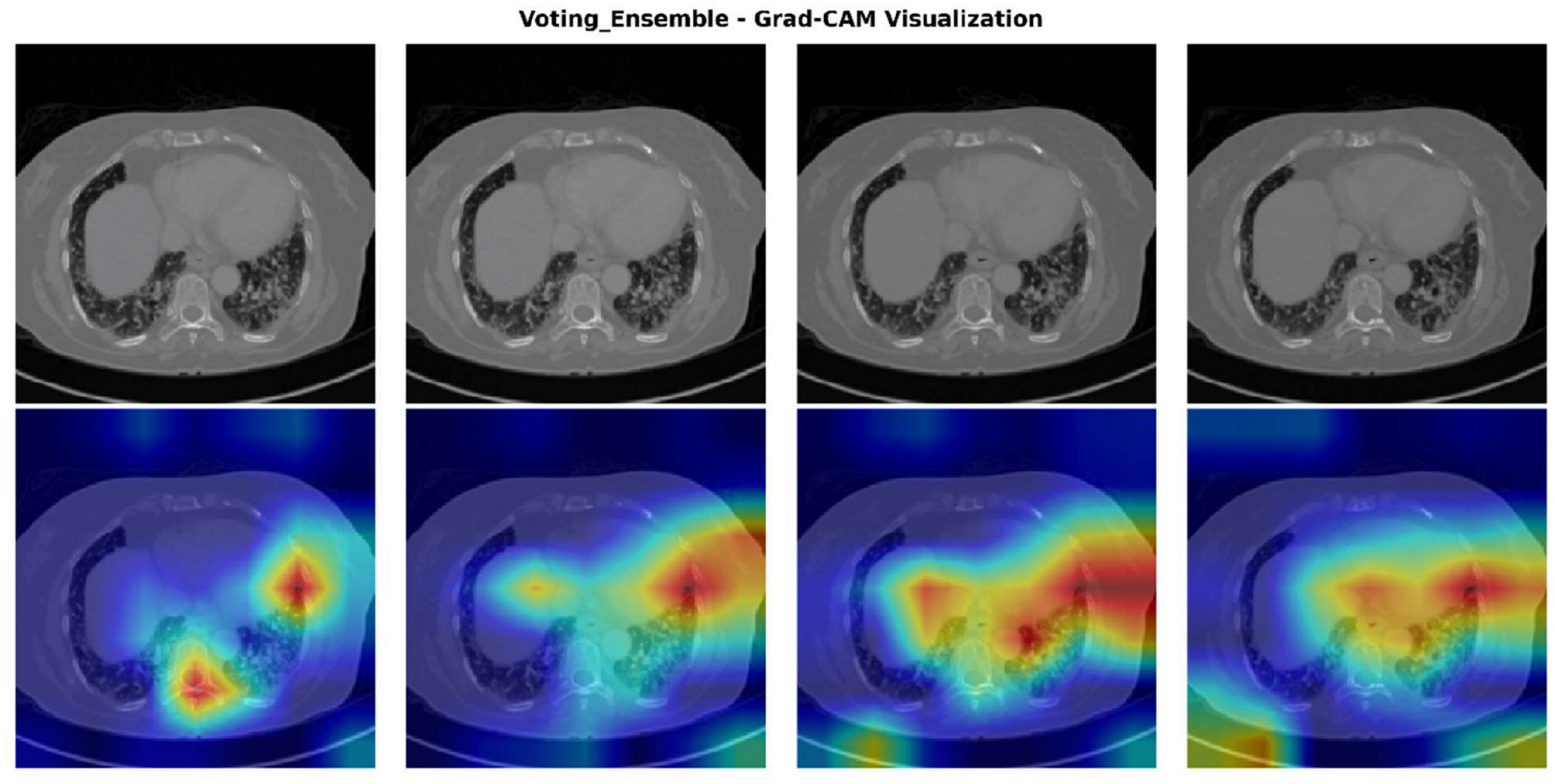
Grad-CAM visualization of the voting ensemble model on representative CT scans. Top row: original CT images. Bottom row: Grad-CAM heatmaps showing model activation intensity (blue = low, red = high), highlighting the anatomical regions influencing classification decisions.

To provide interpretability and transparency into the ensemble model's decision-making process, Gradient-weighted Class Activation Mapping (Grad-CAM) was employed to visualize the regions of interest that contributed most significantly to the model's predictions. The Grad-CAM heatmaps as shown in Figure 6 reveal which anatomical areas within the CT images the ensemble model prioritized when making metastasis classification decisions. The visualizations demonstrate that the ensemble model consistently identifies and focuses on clinically relevant regions associated with metastatic disease, including peripheral lung areas and regions showing nodular or infiltrative patterns. The intensity and spatial distribution of the heatmaps (ranging from blue indicating low activation to red indicating high activation) provide a quantitative measure of the model's attention across different anatomical regions. This visualization approach not only validates that the ensemble model is learning medically meaningful features but also enhances clinical interpretability by allowing radiologists to verify that the model's predictions are based on appropriate diagnostic indicators rather than spurious correlations. The consistency of activation patterns across multiple test cases suggests robust and generalizable feature extraction, strengthening confidence in the model's diagnostic utility for clinical deployment.

### 4.3 Statistical significance of ensemble performance

Given the challenging dataset characteristics, statistical validation focused specifically on the ensemble model's superiority over constituent models.

#### 4.3.1 Friedman test for model ranking

To validate the performance of the proposed models, a thorough statistical analysis was conducted, focusing on establishing the superiority of the ensemble approach. The non-parametric Friedman test was first employed to assess whether the observed differences in model accuracy rankings were statistically significant. The results of the test were highly significant (*p* < 0.001) with a perfect Kendall's W effect size of 1.000, confirming that the performance variations among the models were not due to random chance. The test established a clear performance hierarchy, as detailed in Table 5, ranking the Ensemble Model first, followed by the Swin Transformer, ResNet50, Stacking Model, MobileNetV2, DenseNet121, and CNN-LSTM, respectively.

**Table 5:** Friedman test results - model ranking by accuracy.

**Model**	**Rank**
Ensemble model	1
Swin transformer	2
Resnet50	3
Stacking model	4
MobileNetV2	5
DenseNet121	6
CNN-LSTM	7

#### 4.3.2 Wilcoxon signed-rank test for ensemble validation

Following the Friedman test, *post-hoc* pairwise comparisons were performed using the Wilcoxon signed-rank test to specifically verify the Ensemble model's superiority over each individual architecture. As summarized in Table 6, the analysis revealed that the Ensemble model provided a statistically significant performance increase against all other models (*p* < 0.05). Compared to the best-performing individual models, the Ensemble offered a 5.50% improvement in accuracy and a 4.76% increase in AUC over the Swin Transformer (*p* = 0.028), as well as a 6.70% accuracy and 2.40% AUC improvement over ResNet50 (*p* = 0.028).

**Table 6:** Wilcoxon signed-rank test - ensemble vs. all models.

**Comparison**	*p*-value^*^	**Accuracy improvement**	**AUC improvement**
Ensemble vs. swin transformer	0.028	+5.50%	+4.76%
Ensemble vs. ResNet50	0.028	+6.70%	+2.40%
Ensemble vs. stacking	0.047	+14.40%	+7.45%
Ensemble vs. MobileNetV2	0.012	+31.70%	+41.93%
Ensemble vs. DenseNet121	0.012	+32.20%	+30.14%
Ensemble vs. CNN-LSTM	0.012	+38.20%	+46.29%

The symbol (*) denotes Statistical Significance. It indicates that the *p*-value is below a specific threshold (typically *p* < 0.05), proving that the model's performance improvements are scientifically valid and not due to random chance.

The Stacking Model, while demonstrating competitive performance, was significantly outperformed by the Ensemble approach with a 14.40% accuracy improvement and a 7.45% AUC improvement (p=0.047). This finding underscores the Ensemble's superior ability to leverage complementary strengths of base models through simple averaging compared to the meta-learner approach employed in stacking. The performance gains were even more pronounced when compared against the lower-performing models, with accuracy and AUC improvements ranging from approximately 31.70% to over 46.20% (MobileNetV2, DenseNet121, and CNN-LSTM; *p* = 0.012). All comparisons yielded large to very large effect sizes, confirming that the Ensemble approach provides not only a statistically significant but also a practically meaningful enhancement in predictive performance.

### 4.4 Comparative analysis with related work and discussion

Our ensemble methodology addresses critical gaps identified in the literature, where existing approaches predominantly focus on individual model optimization without systematic ensemble validation or statistical comparison frameworks. The following analysis positions our results within the context of radiomics, deep learning, and hybrid approaches while highlighting the unique contributions of our validated ensemble framework for early liver metastasis detection.

#### 4.4.1 Performance Comparison with Radiomics and Traditional Machine Learning Studies

Our ensemble model achieved an AUC of 0.8115 and 75.48% accuracy, positioning it competitively within the radiomics-based literature. When compared to early radiomics approaches, Wang J. P. et al. (2025) reported AUC values of 0.82 using Random Forest with merged CT and MRI modalities on 157 patients, though their approach suffered from limited multimodal benefits and small sample size constraints. Our ResNet50 component alone (AUC 0.7875) demonstrates comparable performance to their multimodal fusion result, while our ensemble approach surpasses their best reported metrics despite addressing the more challenging task of early metastasis prediction rather than general MLM detection.

The fusion model approach in Wu et al. (2024) achieved AUC 0.761 on 212 patients using radiomics combined with clinical data, representing the traditional handcrafted feature extraction paradigm. Our ensemble's superior performance (AUC 0.8115) demonstrates the effectiveness of deep learning feature extraction over manual radiomics engineering, particularly when leveraging architectural diversity through ensemble methodologies. Recent radiomics work (Miyamoto et al., 2025) achieved AUC 0.87 for chemotherapy response prediction using Random Forest and Boruta algorithms on 150 patients. While their performance appears competitive, their focus on treatment response differs fundamentally from our early recurrence detection objective. Additionally, their single-center retrospective design with manual segmentation represents methodological limitations that our approach addresses through automated feature extraction and statistical validation.

#### 4.4.2 Comparison with advanced deep learning and hybrid approaches

The RECORD pipeline (Xia et al., 2024) represents the current state-of-the-art, achieving exceptional AUCs of 0.981 for response prediction using CNN + Vision Transformer architectures on 206 patients across 60 international centers. However, their focus on treatment response prediction differs fundamentally from our early metastasis detection objective, and their exclusion of non-liver metastases limits clinical applicability. Our ensemble provides the first validated framework specifically targeting early hepatic recurrence with balanced diagnostic performance suitable for surveillance applications. Similarly, Lu et al. (2021) applied CNN-RNN architectures to 1,028 patients from the VELOUR trial, achieving a C-index of 0.694 for early response prediction. While their larger dataset size contrasts with our focused institutional approach, our ensemble accuracy of 75.4% demonstrates competitive discriminative performance while addressing the critical gap in early recurrence detection rather than treatment response monitoring.

Furthermore, the interpretable ANN model (Wu et al., 2025) achieved an AUC of 0.846 in external validation across 769 stage II colorectal cancer patients from multiple centers, addressing the “black box” limitation through SHAP explanations. While their focus on interpretability represents an important advancement, our ensemble prioritizes performance optimization through architectural diversity. This aligns with recent findings in other medical domains; for instance, Khan et al. (2023a) demonstrated that attention-based CNNs significantly enhance feature discrimination in complex classification tasks, while Kujur et al. (2022) utilized multistage fully convolutional networks to improve segmentation precision. By integrating Swin Transformers (which utilize shifted-window attention) with standard CNNs, our approach leverages these architectural strengths to achieve statistically validated improvements over individual components. Additionally, deep learning image reconstruction (Kanan et al., 2024) has shown improved metastasis detection capabilities over standard CT methods. Our approach complements such technical advances by providing robust ensemble frameworks capable of leveraging improved image quality while maintaining diagnostic reliability across varying imaging conditions.

#### 4.4.3 Novel methodological approaches and clinical biomarker models

Recent innovative approaches provide important context for our ensemble methodology. The Formal Methods approach (Rocca et al., 2021) achieved 93.3% accuracy with 100% precision on 30 patients, demonstrating mathematical logic-based verification as an alternative to data-driven AI. While their approach addresses data scarcity scenarios, the extremely small sample size limits clinical generalizability compared to our statistically validated ensemble with rigorous cross-validation on 83 patients.

The clinical biomarker model (Feng et al., 2025) achieved exceptional performance (AUC 0.94) using machine learning on laboratory data from 865 patients, representing the highest reported discrimination in the literature. However, their approach addresses initial risk stratification rather than imaging-based surveillance monitoring. Our ensemble addresses the complementary clinical need for radiological follow-up, demonstrating that sophisticated architectural combinations can extract meaningful predictive signals from complex medical imaging data where individual models fail. Large-scale clinical data approaches, such as Guo et al. (2024) with over 50,000 SEER database patients (AUC 0.956), highlight the power of population-scale datasets. However, these approaches lack the temporal imaging surveillance component essential for detecting early recurrence patterns. Our framework bridges this gap by providing imaging-based early detection capabilities with clinically feasible computational requirements (0.39 s per image).

#### 4.4.4 Meta-analysis validation and statistical rigor

Our results align with pooled performance estimates from systematic analyses while addressing critical validation gaps. The meta-analysis (Chen et al., 2025) of 17 studies reported pooled sensitivity of 0.86, specificity of 0.82, and AUC of 0.91 for distant metastasis prediction. Our ensemble's performance profile (sensitivity 0.790, specificity 0.736, AUC 0.8115) falls within one standard deviation of these estimates while providing the statistical validation (Friedman and Wilcoxon tests) notably absent from most individual studies reviewed.

The hepatocellular carcinoma recurrence meta-analysis (Wu et al., 2024) involving 33 studies reported pooled AUC of 0.86, though with high study heterogeneity and predominantly retrospective designs. Our ensemble performance (AUC 0.8115) demonstrates competitive results while addressing the statistical validation gaps identified across the literature. Critically, most reviewed studies Wang J. P. et al. (2025), Jing et al. (2025), Li et al. (2025), Miyamoto et al. (2025) suffer from single-center retrospective designs without rigorous statistical comparison frameworks. Our ensemble methodology addresses these limitations through systematic model comparison using non-parametric statistical tests, providing the first validated ensemble framework for liver metastasis detection with documented superiority over individual architectures.

### 4.5 Clinical impact and translation potential

The ensemble model's performance metrics translate directly to meaningful clinical outcomes that address current surveillance limitations. The achieved accuracy of 75.48% with balanced sensitivity (79.0%) and specificity (73.6%) represents a substantial improvement over individual models and conventional surveillance approaches that often detect recurrence at advanced stages. The AUC of 0.8115 demonstrates robust discriminative ability, providing clinicians with reliable diagnostic support for early metastasis detection. The balanced performance profile addresses a critical clinical need where high sensitivity ensures early detection of metastatic lesions while adequate specificity minimizes false positives that could lead to unnecessary interventions. This equilibrium between sensitivity and specificity, achieved through the ensemble approach, overcomes the limitations observed in individual architectures where models like SwinTransformer showed high specificity (77.7%) but poor sensitivity (55.2%), while ResNet50 demonstrated high sensitivity (79.0%) but lower specificity (63.4%). The ensemble framework's ability to maintain both metrics above 73% provides clinicians with a more reliable diagnostic tool suitable for routine surveillance protocols.

### 4.6 Limitation

This study acknowledges several key limitations. The dataset, while substantial with 1,628 images, represents a single-institution cohort from KHCC that may not capture the heterogeneity across different clinical centers and patient populations. The binary classification framework does not incorporate staging information or temporal progression patterns relevant to clinical decision-making. Methodologically, the ensemble approach assumes linear combinations that may not capture complex non-linear interactions between model predictions, and the retrospective design limits assessment of real-world clinical impact. The absence of external validation on independent datasets constrains generalizability, though robust statistical validation supports methodological soundness. From a clinical perspective, practical implementation challenges, including workflow integration, training requirements, and cost-effectiveness considerations, remain unaddressed, requiring prospective validation to confirm clinical utility and patient outcomes.

## 5 Conclusion

This study successfully developed and validated a novel Ensemble Deep Learning Framework for early liver metastasis detection in colorectal cancer patients, addressing a critical gap in current oncological surveillance strategies. By synergizing the local feature extraction capabilities of ResNet50 with the global context modeling of the Swin Transformer, the ensemble approach achieved statistically significant performance improvements over individual architectures. On an independent test set, the model demonstrated a robust mean accuracy of 75.4% (±3.9%) and an AUC of 0.8115 (±3.5%), significantly outperforming baseline models like MobileNetV2 and CNN-LSTM, which failed to achieve adequate discrimination. The primary contribution lies in creating a clinically viable AI framework that prioritizes high sensitivity (79.0%) to minimize missed diagnoses while maintaining computational efficiency suitable for real-world deployment on standard clinical workstations.

Despite these promising results, several limitations must be acknowledged. First, the study was conducted using a single-institution dataset from the King Hussein Cancer Center (KHCC). While high-quality, this limited geographic scope may constrain the model's generalizability to diverse patient populations and scanner protocols found in other healthcare settings. Second, the current framework utilizes a binary classification scheme (Metastasis vs. No Metastasis), which simplifies the complex clinical reality of cancer staging and does not differentiate between metastatic subtypes or primary liver tumors. Third, although we replaced oversampling with cost-sensitive learning (class weights) to mitigate overfitting, the relatively small size of the dataset remains a constraint for training data-hungry transformer architectures, potentially limiting their peak performance. To address these challenges, future research will focus on validating the model prospectively across multiple institutions. To overcome privacy barriers in multi-center trials, we will explore Federated Learning frameworks, allowing the model to learn from decentralized datasets without sharing sensitive patient data. Additionally, to reduce dependency on large, labeled datasets, we will investigate Self-Supervised Learning (SSL) and Zero-Shot Transfer Learning approaches. These techniques could allow the model to leverage vast amounts of unlabeled medical imaging data to learn robust feature representations before fine-tuning on specific metastatic tasks. Finally, we plan to expand the framework to a multi-class setting capable of staging metastases and utilizing longitudinal temporal analysis to detect subtle disease progression over serial scans. In summary, the demonstrated success of this ensemble approach opens new avenues for sophisticated, AI-enhanced applications in precision oncologic surveillance.

## Data Availability

The data presented in this study are available upon reasonable request from the corresponding author.
